# Interpretable multiphasic CT-based radiomic analysis for preoperatively differentiating benign and malignant solid renal tumors: a multicenter study

**DOI:** 10.1007/s00261-024-04351-3

**Published:** 2024-05-11

**Authors:** Yaohai Wu, Fei Cao, Hanqi Lei, Shiqiang Zhang, Hongbing Mei, Liangchao Ni, Jun Pang

**Affiliations:** 1 Department of Urology, The Seventh Affiliated Hospital, Sun Yat-sen University, Shenzhen, China; 2 Department of Urology, Shenzhen Second People’s Hospital, The First Affiliated Hospital of Shenzhen University, Shenzhen, China; 3 Department of Urology, Guangdong and Shenzhen Key Laboratory of Reproductive Medicine and Genetics, Peking University Shenzhen Hospital, Shenzhen, China

**Keywords:** Computed tomography, Radiomics, Random forest, Renal tumors, SHapley Additive exPlanations

## Abstract

**Background:**

To develop and compare machine learning models based on triphasic contrast-enhanced CT (CECT) for distinguishing between benign and malignant renal tumors.

**Materials and Methods:**

In total, 427 patients were enrolled from two medical centers: Center 1 (serving as the training set) and Center 2 (serving as the external validation set). First, 1781 radiomic features were individually extracted from corticomedullary phase (CP), nephrographic phase (NP), and excretory phase (EP) CECT images, after which 10 features were selected by the minimum redundancy maximum relevance method. Second, random forest (RF) models were constructed from single-phase features (CP, NP, and EP) as well as from the combination of features from all three phases (TP). Third, the RF models were assessed in the training and external validation sets. Finally, the internal prediction mechanisms of the models were explained by the SHapley Additive exPlanations (SHAP) approach.

**Results:**

A total of 266 patients with renal tumors from Center 1 and 161 patients from Center 2 were included. In the training set, the AUCs of the RF models constructed from the CP, NP, EP, and TP features were 0.886, 0.912, 0.930, and 0.944, respectively. In the external validation set, the models achieved AUCs of 0.860, 0.821, 0.921, and 0.908, respectively. The “original_shape_Flatness” feature played the most important role in the prediction outcome for the RF model based on EP features according to the SHAP method.

**Conclusions:**

The four RF models efficiently differentiated benign from malignant solid renal tumors, with the EP feature-based RF model displaying the best performance.

**Graphical Abstract:**

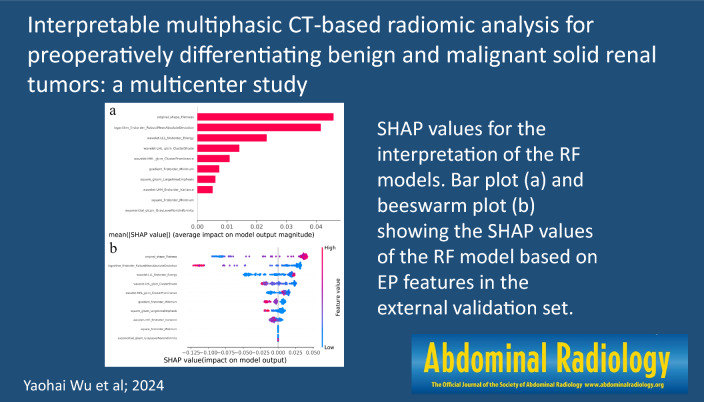

**Supplementary Information:**

The online version contains supplementary material available at 10.1007/s00261-024-04351-3.

## Introduction

Renal cancer is the third leading cause of genitourinary cancer death, and its incidence has been increasing over the past decade [1]. Renal cell carcinoma (RCC) constitutes approximately 70% of all renal cancers; its most common histopathological subtypes include clear cell RCC (ccRCC), papillary RCC (pRCC), and chromophobe RCC (chRCC) [2]. Patients diagnosed with renal cancer are recommended to undergo radical or partial nephrectomy as soon as possible [3]. However, in clinical practice, for some patients with benign renal tumors, including renal oncocytoma (RO) and angioleiomyolipoma (AML), cannot be accurately distinguished from malignant renal tumors, including ccRCC, pRCC and chRCC, with imaging before surgery [4, 5]. RO, a benign tumor constituting 3–5% of renal neoplasms in adults, can be misclassified as RCC on imaging; this misclassification is responsible for 4–10% of surgeries performed for suspected RCC [4]. Additionally, as many as 5% of renal AML exhibit insufficient fat content, making differentiation from RCC using conventional computed tomography (CT) and magnetic resonance imaging (MRI) techniques challenging [5]. Consequently, patients with benign renal tumors can be misdiagnosed with malignant renal tumors, potentially resulting in surgical treatment that may impose surgical complications and a heavy economic burden. Therefore, it is highly important to find a simple, noninvasive and accurate method for preoperatively differentiating benign from malignant solid renal tumors.

Developments in medical examination techniques have led to CT, ultrasound and MRI playing increasingly important roles in the differential diagnosis of tumors [6, 7]. In clinical practice, CT is an important method for the differential diagnosis of renal tumors because it is more precise than ultrasound and more convenient than MRI [8, 9]. While the value of CT imaging in the differential diagnosis of renal tumors continues to increase [10, 11], importantly, its performance in differentially diagnosing renal tumors remains less than ideal. Renal tumor biopsy is another important tool used for histological diagnosis, with a diagnostic rate of 78–97% for malignancies [12]. However, despite this importance, renal biopsy presents with inherent risks such as bleeding, seeding and pain [13]. Moreover, due to the limited tissue samples obtained during biopsy, there is a risk of incomplete tumor representation, leading to a potentially inaccurate analysis. Additionally, both renal imaging reports and biopsy results heavily rely on the proficiency of the medical practitioner or operator, impacting their accuracy. Therefore, the pursuit of a less invasive yet highly accurate method for renal tumor differentiation is imperative.

Radiomics can very efficiently extract large-scale imaging features from medical images, including first-order features and transformed filter-based features that cannot be identified by the naked eye, turning imaging data into a high-resolution mineable resource for guiding clinical practice [14–17]. Many studies have reported that radiomics plays an important role in solid tumor diagnosis, biological characterization and prognosis prediction, and decision-making assistance [18, 19]. Moreover, several studies have shown that CT-based radiomics combined with machine learning (ML) has important advantages for renal tumor differentiation and risk and prognosis prediction [20–22]. Thus, it is important to investigate the utility of radiomics for improving and optimizing the CT-based differential diagnosis of renal tumors.

Although the aforementioned studies successfully constructed adequate radiomic-based models, they did not elucidate the influence of internal features on model performance. Nevertheless, it is imperative for a robust prediction model to possess the capacity for interpreting its inherent “black box” nature, a consideration of great significance. To address the challenge posed by the “black box” nature, the SHapley Additive exPlanations (SHAP) method was introduced to help clinicians understand the results of constructed models [23]. The sign of the SHAP value of a feature signifies the direction in which that feature exerts its influence, while the magnitude of the SHAP value denotes the “weight” or “significance” of the respective feature. Consequently, a number of researchers have embraced the SHAP method as a means to clarify the influence of radiomic features used for model construction on both overall and individual prediction outcomes [24–27].

Consequently, the primary objective of this study was to establish and evaluate radiomic models incorporating ML algorithms to differentiate between benign and malignant solid renal tumors via contrast-enhanced CT (CECT) scans. Furthermore, the SHAP model was employed to interpret our model results.

## Materials and methods

### Patients

This retrospective study received approval from the institutional review board (KY-2023–083-01), who waived the need for written informed consent.

**Fig. 1:**
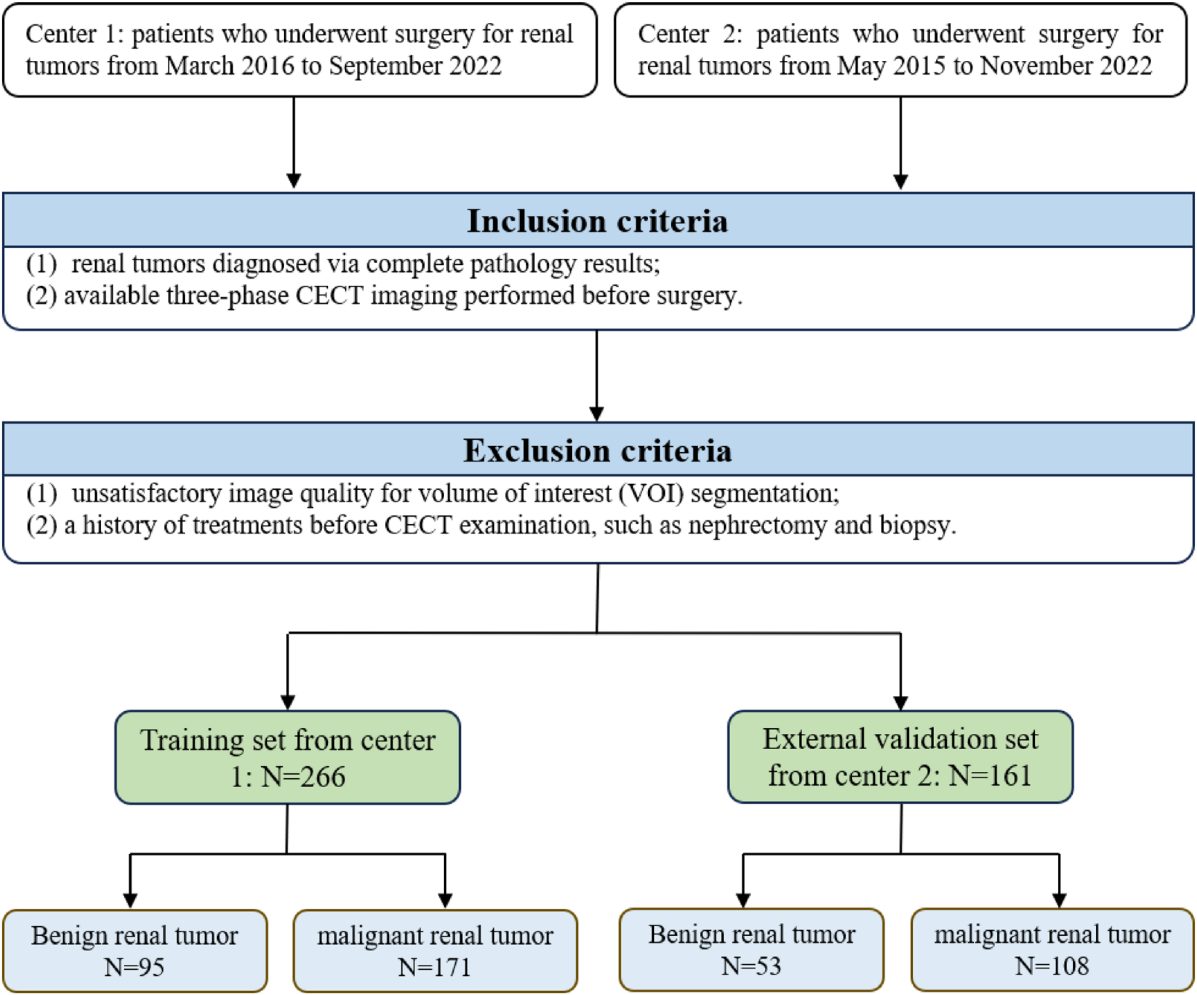
Flow diagram for selection of the study population

The training set for this retrospective study was compiled from Center 1, spanning from May 2016 to September 2022, while the external validation set was sourced from Center 2, encompassing the period from August 2013 to November 2021. The inclusion criteria were as follows: (1) renal tumors diagnosed via complete pathology results and (2) available three-phase CECT imaging performed before surgery. The exclusion criteria were as follows: (1) unsatisfactory image quality for volume of interest (VOI) segmentation and (2) a history of treatments before CECT examination, such as nephrectomy and biopsy. A schematic representation of the study population is provided in Fig. 1. Given the greater ease with which T3 RCC can be differentiated from any benign renal mass than T1a RCC can, we also selected patients with renal tumors measuring ≤ 4 cm in diameter for subanalysis.

### Image acquisition and tumor segmentation

Axial three-phase scanning, encompassing the corticomedullary phase (CP), nephrographic phase (NP), and excretory phase (EP), was conducted for each patient using multislice spiral CT scanners. Supplementary Table 1 displays the CECT protocols used in this study. Renal CECT images were downloaded from the local medical image management system of the respective hospitals. The three-dimensional (3D) VOI of the renal tumor was manually delineated on the axial CP, NP, and EP images using ITK-SNAP 3.8.0 by a junior urologist (YH W, with 5 years of experience) and a senior urologist (SQ Z, with more than 10 years of experience). All VOIs were subsequently examined by a senior radiology professor (with more than 20 years of experience) and a senior urology professor (J P, with more than 20 years of experience). The VOI was manually outlined along the outermost boundaries of the tumor, with care taken to avoid adjacent normal tissue. If the patient had multiple renal tumors on one side, only the one with the largest diameter was outlined.

### Feature extraction

A total of 1781 radiomic features were extracted from the 3D VOIs of the renal tumors on each set of CECT phase images using PyRadiomics (version 3.0.1) in Python 3.7.6. Voxel resampling and gray discretization were conducted to make the different pixel spacings of the CECT images from different CT scanners uniform between the patients in the two centers before feature extraction [28–30]. The images were resampled to a 1 × 1 × 1 mm^3^ voxel size using nearest neighbor interpolation, and the bin width of gray discretization was set to 25. The extracted radiomic features were normalized utilizing the Z score method after feature extraction [31].

### Feature selection

The minimum redundancy maximum relevance (mRMR) method was used to select the 10 most representative features. This algorithm can ensure the selection of highly relevant features with respect to real categories while simultaneously eliminating redundancy among the selected features [32–34]. Agglomerative hierarchical clustering was employed to generate a cluster heatmap illustrating disparities in the radiomic features selected from the CP, NP, and EP images between the malignant and benign solid renal tumor groups and to visualize the relationships between subject clusters and these selected radiomic features.

### Model construction and evaluation

**Fig. 2:**
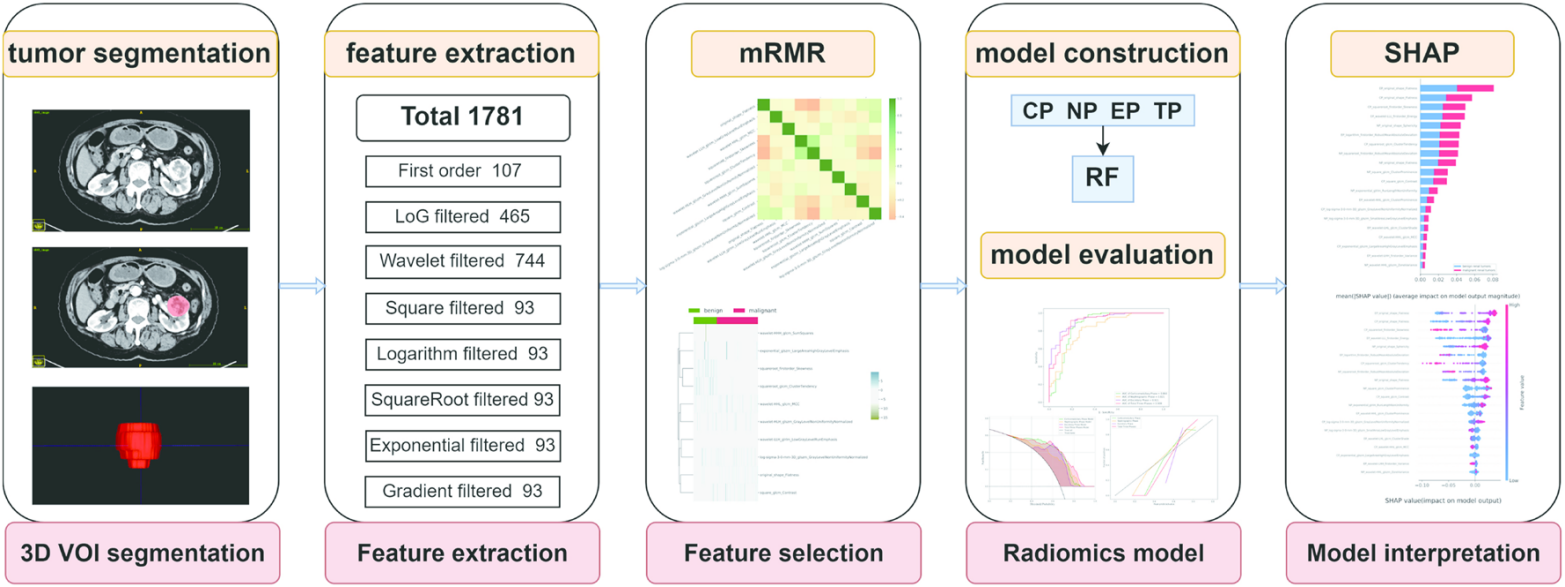
Flow diagram of model construction and interpretation in this study

Subsequently, random forest (RF) models were constructed using the Scikit-learn library version 1.0.2, employing the selected 10 features. The RandomizedSearchCV method was individually utilized to fine-tune the hyperparameters of the constructed models. A summary of the ML model development workflow is provided in Fig. 2.

Four RF models were constructed utilizing selected representative features derived from both the single phases (CP, NP, and EP) and the combination of all three phases (TP). The RF model based on TP features was constructed using the combination of the 10 features selected from the CP images, the 10 features selected from the NP images, and the 10 features selected from the EP images. These models were subsequently applied to the external validation set for evaluation. Model performance was evaluated through receiver operating characteristic (ROC) curve analysis. Additionally, metrics including the area under the ROC curve (AUC), accuracy, sensitivity, and specificity were computed for each model.

### Model interpretation

SHAP was employed to interpret the importance of the radiomic features incorporated within the best RF model. SHAP enables the visualization of feature importance within intricate ML-based models, helping elucidate how individual features within the model impact the likelihood of a particular output either positively or negatively.

### Statistical analysis

Differences in demographic characteristics between the training set and the external validation set were evaluated using R 4.3.0. The χ^2^ test was employed for comparing categorical variables, while the Mann‒Whitney U test was applied for comparing nonnormally distributed continuous variables. A significance level of *P* < 0.05 indicated statistical significance [35].

## Results

### Clinical characteristics of the study population

In this study, 427 patients were enrolled. A total of 266 patients from Center 1 were included in the training set, and 161 patients from Center 2 were included in the external validation set. No significant differences were observed in the clinical characteristics (age, sex, laterality, tumor classification, or tumor size) between the training set and the external validation set. The detailed clinical characteristics of the study population are provided in Table 1. The detailed histologic distribution of renal tumors is described in Supplementary Table 2. The detailed stages and grades of RCC tumors are provided in Supplementary Table 3. The detailed distribution of benign and malignant renal tumors in patients with tumors measuring ≤ 4 cm in diameter is shown in Supplementary Table 4.

**Table 1:** Demographic characteristics of the study population

Characteristics		Training set (N = 266)	External validation set (N = 161)	*P*
Age (median [IQR])		49.5 [40.2, 59.0]	51.0 [42.0, 61.0]	0.263^*^
Sex (%)	Female	137 (51.50)	77 (47.83)	0.524^**^
	Male	129 (48.50)	84 (52.17)	
Laterality (%)	Left	146 (54.89)	86 (53.42)	0.845^**^
	Right	120 (45.11)	75 (46.58)	
Tumor classification (%)	Benign	95 (35.71)	53 (32.92)	0.629^**^
	Malignant	171 (64.29)	108 (67.08)	
Size (median [IQR])		4.2 [3.1, 6.0]	4.0 [2.8, 5.5]	0.199^*^

Note. The median (interquartile range, IQR) is shown for nonnormally distributed continuous variables. The number of patients (percentage, %) is shown for categorical variables

^*^P values were calculated utilizing the Mann‒Whitney U test

^**^P values were computed using χ^2^ tests

### Features selected by mRMR

**Fig. 3:**
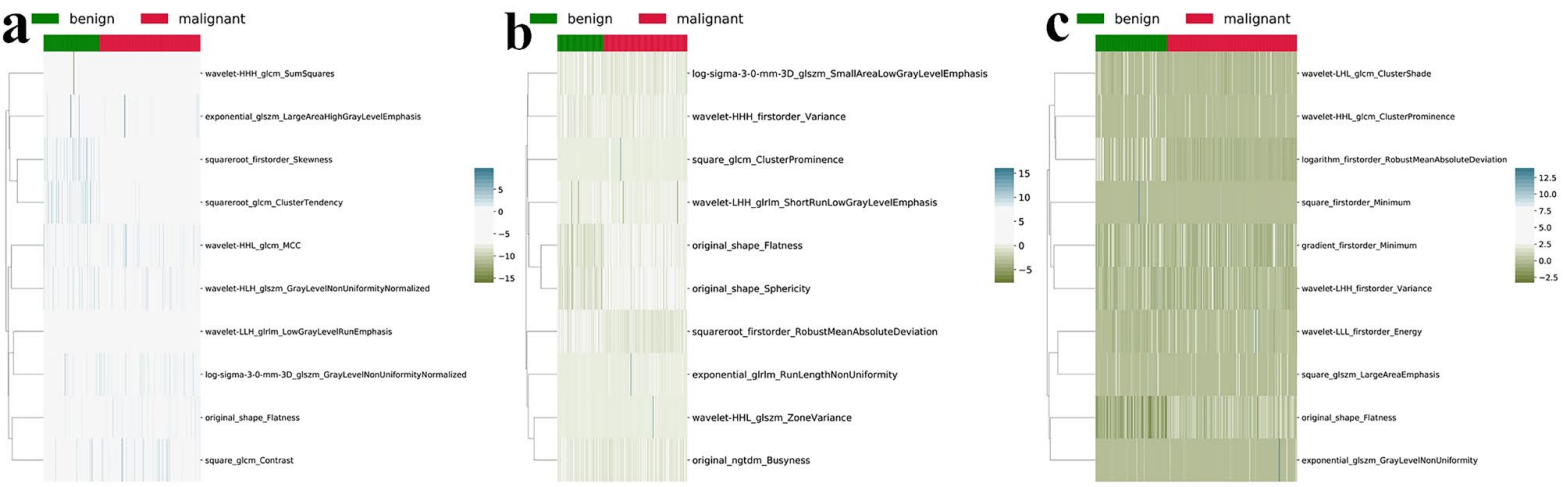
Cluster heatmap of the 10 most representative features extracted from CP (**a**), NP (**b**) and EP features (**c**)

The details of the 10 features selected by the mRMR method from the CP, NP, and EP images are provided in Supplementary Table 5. A cluster heatmap illustrating differences in radiomics features selected from CP, NP, and EP images between the malignant and benign renal tumor groups is shown in Fig. 3. The three most distinct features between the malignant and benign renal tumor groups are summarized in Table 2.

**Table 2:** The three most distinct features between the malignant and benign renal tumor groups for each phase of images

Phase	Feature name	Change
CP	original_shape_Flatness	higher
	squareroot_firstorder_Skewness	lower
	squareroot_glcm_ClusterTendency	lower
NP	squareroot_firstorder_RobustMeanAbsoluteDeviation	lower
	original_shape_Sphericity	higher
	original_shape_Flatness	higher
EP	original_shape_Flatness	higher
	logarithm_firstorder_RobustMeanAbsoluteDeviation	lower
	wavelet-LLL_firstorder_Energy	higher

Note. The term ‘Change’ denotes the change in the feature value in the malignant group with respect to the benign group

### RF model construction and evaluation

The RF models were constructed according to the hyperparameters derived with the training set. Detailed hyperparameter information is available in Supplementary Table 6.

The AUC, accuracy, specificity and sensitivity of the RF model based on CP were 0.886 (0.848–0.924), 0.838 (0.794–0.883), 0.832 (0.787–0.877) and 0.801 (0.753–0.849), respectively, in the training set. The AUC, accuracy, specificity and sensitivity of the RF model based on NP images were 0.912 (0.878–0.946), 0.842 (0.798–0.886), 0.737 (0.684–0.790) and 0.930 (0.899–0.961), respectively, in the training set. The AUC, accuracy, specificity and sensitivity of the RF model based on EP images were 0.930 (0.900–0.961), 0.786 (0.736–0.835), 0.821 (0.775–0.867) and 0.883 (0.844–0.922), respectively, in the training set. The AUC, accuracy, specificity and sensitivity of the RF model based on TP were 0.944 (0.916–0.972), 0.868 (0.828–0.909), 0.821 (0.775–0.867) and 0.906 (0.871–0.941), respectively, in the training set.

The constructed RF models based on CP, NP, EP, and TP features yielded AUC values of 0.860 (0.806–0.914), 0.821 (0.762–0.880), 0.921 (0.879–0.963), and 0.908 (0.864–0.953), respectively, in the external validation set. For the RF model based on CP features, the sensitivity, specificity, and accuracy were 0.889 (0.840–0.937), 0.774 (0.709–0.838), and 0.857 (0.803–0.911), respectively. The RF model based on NP features achieved sensitivity, specificity, and accuracy values of 0.843 (0.786–0.899), 0.717 (0.647–0.787), and 0.789 (0.726–0.852), respectively. Similarly, the RF model based on EP features exhibited good sensitivity (0.926 (0.885–0.966)), specificity (0.774 (0.709–0.838)), and accuracy (0.764 (0.698–0.830)). The RF model based on TP features achieved a sensitivity, specificity, and accuracy of 0.917 (0.874–0.959), 0.811 (0.751–0.872), and 0.826 (0.768–0.885), respectively.

**Fig. 4:**
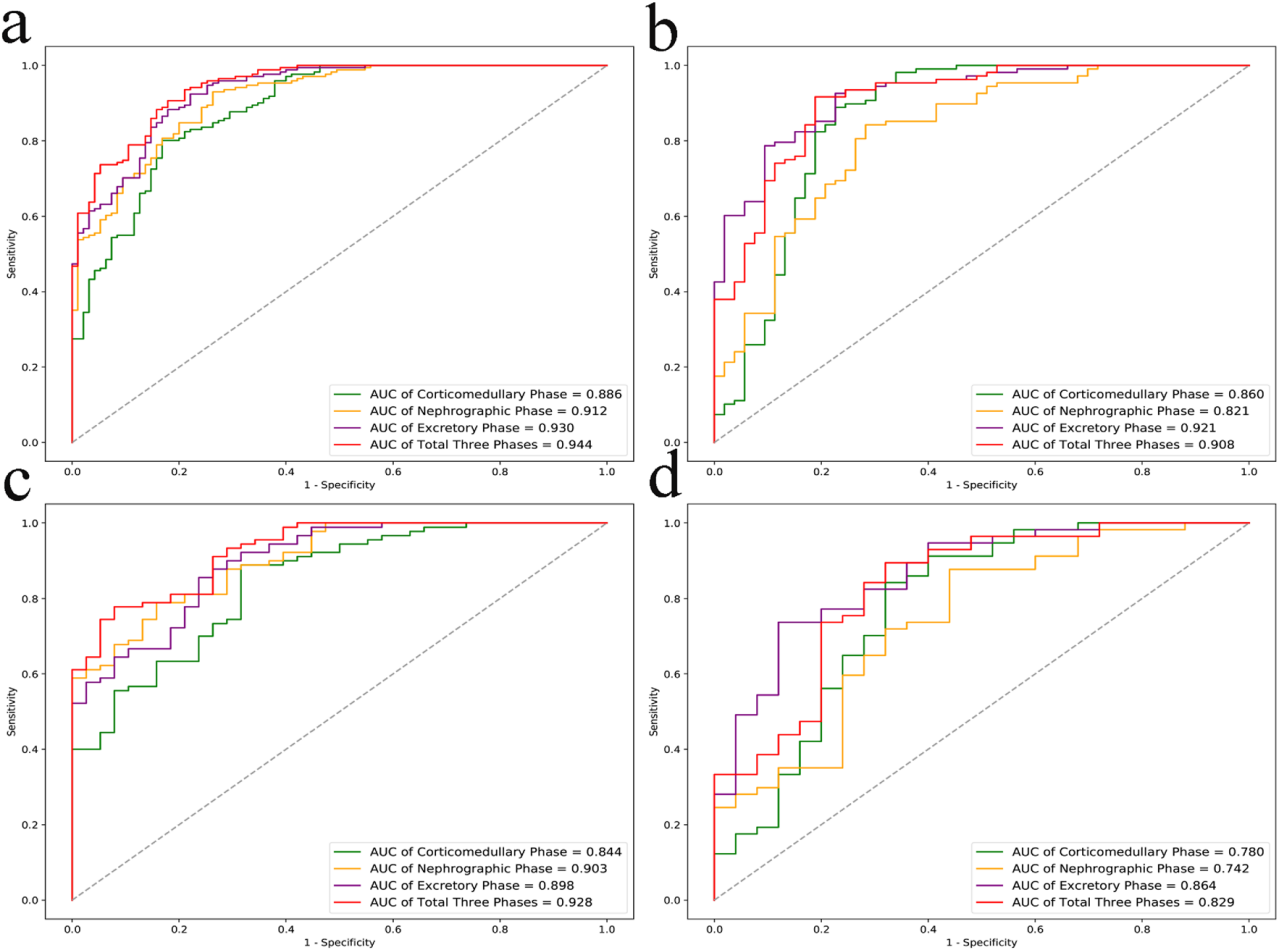
ROC curves of the RF models for all benign and malignant renal tumors in the training set (**a**) and external validation set (**b**). ROC curves of the RF models for benign and malignant renal tumors measuring ≤ 4 cm in diameter in the training set (**c**) and external validation set (**d**)

Finally, the AUC of the RF model based on EP features (0.921) exceeded that based on CP (0.860), NP (0.821), and TP features (0.908) in the external validation set. The predictive performance of the four RF models is summarized in Table 3. The ROC curves, visualization of the results of decision curve analysis, and calibration curves of the RF models based on CP, NP, EP, and TP features are shown in Fig. 4 and Supplementary Fig. 1. The predictive performance of the four RF models for renal tumor patients with tumors measuring ≤ 4 cm in diameter is summarized in Supplementary Table 7. The ROC curves of the RF models based on CP, NP, EP, and TP features for renal tumor patients with tumors measuring ≤ 4 cm in diameter are also shown in Fig. 4. Our findings indicate that the performance of the best model based on EP features for distinguishing T1a RCC has the best AUC of 0.864 in the external validation set.

**Table 3:** Performance of RF models in the training set and external validation set

Metric		AUC	ACC	Spe	Sen	PPV	NPV
Training set	CP	0.886 (0.848–0.924)	0.838 (0.794–0.883)	0.832 (0.787–0.877)	0.801 (0.753–0.849)	0.895 (0.859–0.932)	0.699 (0.644–0.754)
	NP	0.912 (0.878–0.946)	0.842 (0.798–0.886)	0.737 (0.684–0.790)	0.930 (0.899–0.961)	0.864 (0.823–0.905)	0.854 (0.811–0.896)
	EP	0.930 (0.900–0.961)	0.786 (0.736–0.835)	0.821 (0.775–0.867)	0.883 (0.844–0.922)	0.899 (0.863–0.935)	0.796 (0.747–0.844)
	TP	0.944 (0.916–0.972)	0.868 (0.828–0.909)	0.821 (0.775–0.867)	0.906 (0.871–0.941)	0.901 (0.865–0.937)	0.830 (0.785–0.875)
External validation set	CP	0.860 (0.806–0.914)	0.857 (0.803–0.911)	0.774 (0.709–0.838)	0.889 (0.840–0.937)	0.889 (0.840–0.937)	0.774 (0.709–0.838)
	NP	0.821 (0.762–0.880)	0.789 (0.726–0.852)	0.717 (0.647–0.787)	0.843 (0.786–0.899)	0.858 (0.805–0.912)	0.691 (0.620–0.762)
	EP	0.921 (0.879–0.963)	0.764 (0.698–0.830)	0.774 (0.709–0.838)	0.926 (0.885–0.966)	0.893 (0.845–0.941)	0.837 (0.780–0.894)
	TP	0.908 (0.864–0.953)	0.826 (0.768–0.885)	0.811 (0.751–0.872)	0.917 (0.874–0.959)	0.908 (0.864–0.953)	0.827 (0.768–0.885)

Note. The data in brackets are the 95% CIs of the corresponding values

### Enhancing model interpretability using SHAP values

**Fig. 5:**
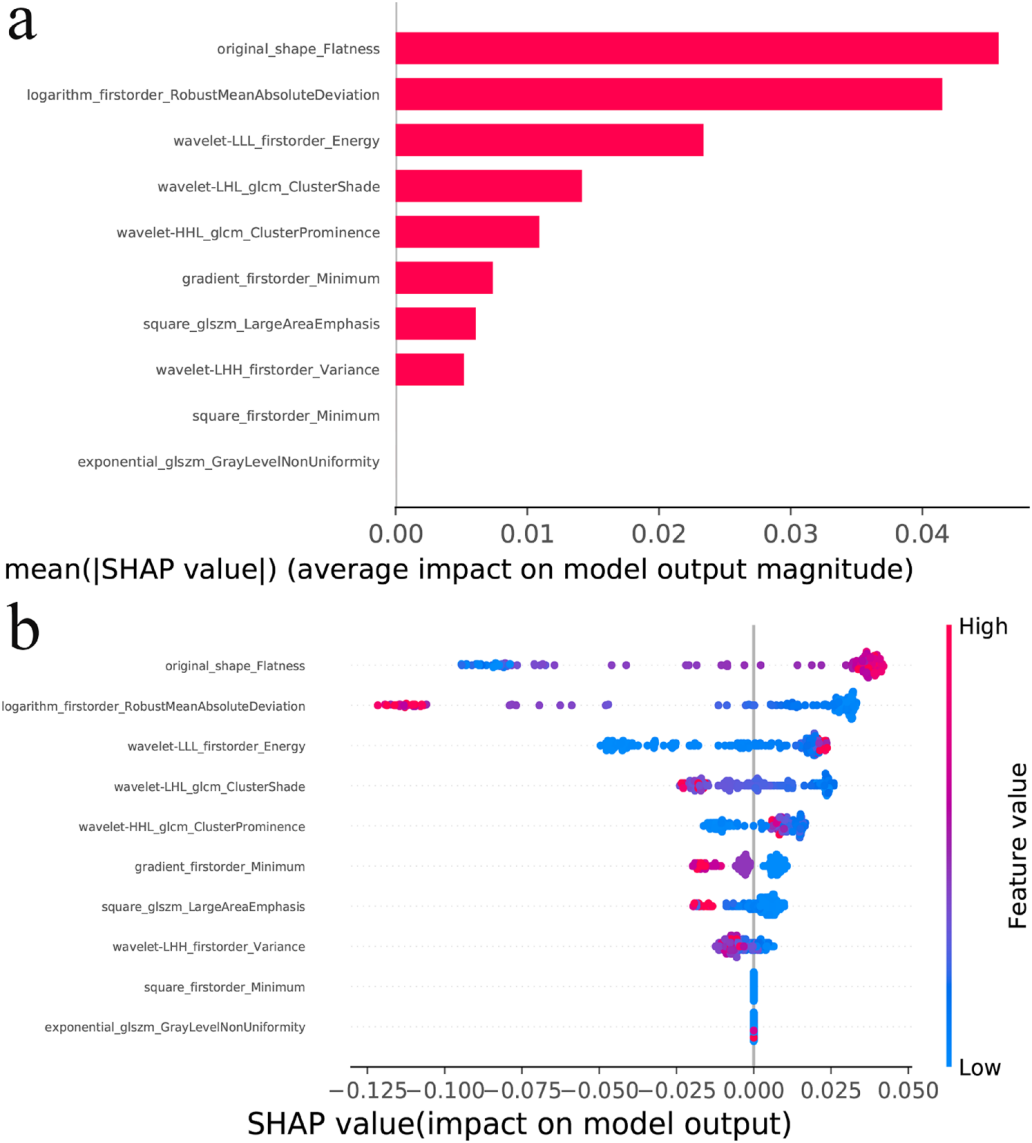
SHAP values for the interpretation of the RF models. Bar plot (**a**) and beeswarm plot (**b**) showing the SHAP values of the RF model based on EP features in the external validation set

The SHAP bar plot, which lists the most crucial features influencing the model output in descending order, and the SHAP beeswarm plot, illustrating the impacts of the features on RF model decisions and the interactions between these features in the model, are shown in Fig. 5. As depicted in the bar plots, the top feature, “original_shape_Flatness”, exhibited the greatest contribution to the model output and possessed stronger predictive capabilities than did the lower-ranked features. As demonstrated in the beeswarm plots, a positive SHAP value for “original_shape_Flatness” corresponds to an increased likelihood of a malignant renal tumor for each prediction, while a negative value indicates the opposite. The magnitude of the value is directly proportional to the (increased or decreased) risk of malignancy. The top three feature importance rankings of the RF model based on EP features are summarized in Table 4.

**Table 4:** The most influential features according to the SHAP method

Phase	SHAP	Feature name	Change	Significance
EP	top 1	original_shape_Flatness	higher	increased risk
	top 2	logarithm_firstorder_RobustMeanAbsoluteDeviation	lower	decreased risk
	top 3	wavelet-LLL_firstorder_Energy	higher	increased risk

Note. The column ‘Change’ denotes the change in SHAP value in the malignant group with respect to the benign group. The column ‘Significance’ denotes that a change in the feature direction results in an increased or decreased risk of malignancy

## Discussion

In this study, four RF models were developed based on CP, NP, EP, and TP features to preoperatively distinguish between benign and malignant renal tumors in patients who underwent CECT scans for solid renal tumor assessment. The RF models were constructed with the features from the training set, and their discriminative capabilities were demonstrated in an external validation set, achieving AUC values ranging from 0.821 to 0.921 and accuracies ranging from 76.4% to 85.7%. Among these models, the RF model based on EP image features exhibited the highest diagnostic performance in the external validation set, with an AUC of 0.921. These results indicate that ML-based radiomic models could offer a straightforward, noninvasive, and highly accurate approach for preoperatively predicting whether a solid renal tumor is benign or malignant with CECT images.

Previous investigations have assessed the potential of radiomic models based on CECT, MRI, and ultrasound for distinguishing benign from malignant renal tumors [36–40]. Nevertheless, these studies exhibited limitations that we aimed to address in this study. Alhussaini et al. concluded that ML-based radiomic analysis holds substantial promise for effectively distinguishing chRCC from RO when employing CECT images (37 chRCC and 41 RO) [38]. Their model yielded the best AUC of 1.00, but the dataset was small, and external validation was lacking. Wang et al. randomly divided 190 RCC patients (147 ccRCC patients and 43 nonccRCC patients) from one center into two groups (a training set and a testing set at a ratio of 7:3) and combined three ML algorithms to develop radiomic models, which demonstrated promising potential in differentiating ccRCC patients from nonccRCC patients [39]. The best AUC achieved by their models was 0.909, but again, the dataset was small, and external validation was lacking. Moreover, when patients with renal tumors seek medical attention, their primary concern is whether the tumor is benign or malignant. Consequently, the most critical aspect is the preoperative differential diagnosis of their renal tumors. Wentland et al. identified 148 solid renal tumors (50 benign: 23 AML, 27 RO; 98 malignant: 23 ccRCC, 44 pRCC, 31 chRCC) and parsed them into two sets (training/testing at a ratio of 7:3) to construct an RF model based on CECT images, which achieved an AUC of 0.80 (with a superior accuracy of 0.82) in distinguishing benign from malignant renal tumors, outperforming 3 radiologists (accuracies from 0.67 to 0.75) [36]. Although the topic of the study was the differentiation of benign and malignant renal tumors, the dataset was still small and lacked an external validation set, and the constructed model lacked interpretability. Massa’a et al. included 182 renal tumors in 160 patients and divided them into training (70%) and testing (30%) sets to assess the performance of ML models trained with MRI-based radiomic features in distinguishing benign from malignant solid renal masses. The model, based on T2-weighted MR images, achieved a high level of accuracy (0.80, with an AUC of 0.79); however, the dataset was still small and lacked an external validation set, and the constructed model lacked interpretability. [37]. Erdim et al. included 79 patients with 84 solid renal tumors and combined 8 ML algorithms to distinguish between benign and malignant renal tumors [40]. In that study, the best model had an AUC of 0.916 and an accuracy of 0.917, which suggested that CT radiomics, in conjunction with ML algorithms, has utility in the noninvasive discrimination of benign and malignant renal tumors. However, the dataset was still small and lacked an external validation set, and the constructed model lacked interpretability. In summary, the abovementioned studies only involved relatively small sample sizes, typically involving fewer than 160 patients, and often lacked an independent validation set and model interpretation. Consequently, the generalizability of the aforementioned research findings may be limited; additional efforts to enhance generalizability would necessitate larger sample sizes, including an independent external validation set, and model interpretation.

It is challenging for clinicians to determine how models draw conclusions and to identify radiomic features that are critical in decision making. In our research, we found that original_shape_Flatness, a 3D shape feature, was the most important feature in informing the prediction outcome of the model according to the SHAP method. The term "flatness" is employed to describe the geometric characteristics of an object, specifically referring to the ratio between its width and length. More precisely, it can be defined as the ratio between the maximum diameter of the target and the minimum diameter perpendicular to it. The values for flatness range from 0 (indicating a perfectly flat shape) to 1 (representing a nonflat, spherical shape). The original_shape_Flatness eigenvalues are computed based on the morphology of the original VOI. In this study, we observed that malignant tumors exhibited greater values for original_shape_Flatness than did benign tumors. This finding aligns with clinical practice, where irregularly shaped tumors are typically considered malignant based on CECT imaging results.

Our research exhibits several strengths to previous studies. First, our study had a larger sample size (n = 427) than the investigations described above. Moreover, we incorporated an external validation dataset (n = 161) to validate the models developed from our training set (n = 266). Second, we harnessed radiomic features extracted from three distinct phases of CECT imaging—CP, NP, and EP imaging—to construct three RF models. Additionally, a fourth RF model was formulated by amalgamating the radiomic features across all phases, denoted as the TP model. The most significant advantage of this study lies in the utilization of the mRMR method for feature selection, coupled with the use of SHAP values to improve model interpretability. The utilization of the mRMR method ensures that the chosen features exhibit the highest correlations with the actual classifications while minimizing redundancy. Notably, the most distinctive features selected by the mRMR method align with the top features identified by SHAP as exerting the greatest influence on the prediction outcomes. As a result of these aforementioned strengths, our research findings possess greater generalizability and practicality.

Although the findings presented in this study provide promising insights into distinguishing renal tumors, several limitations must be acknowledged. First, the retrospective nature of this study introduced inherent selection bias, and the sample size was insufficiently large. Consequently, future research should consider conducting a multicenter study with a larger population to further assess the proposed model. Additionally, prospective studies could be conducted to confirm the robustness of the proposed model. Second, all VOIs in this study were manually segmented, which is a time-intensive process. The use of automatic segmentation techniques is warranted to enhance segmentation efficiency and reduce labor costs in the future.

In summary, all four RF models efficiently differentiated benign from malignant solid renal tumors, and the RF model based on EP features displayed the best performance. The feature “original_shape_Flatness” played the greatest role in predicting the outcome of RF model based on EP image features.

### Supplementary Information

Below is the link to the electronic supplementary material.

Supplementary file1 (DOCX 307 KB)
