# Supplementary material for: Interpretable multiphasic CT-based radiomic analysis for preoperatively differentiating benign and malignant solid renal tumors: a multicenter study
Source: Abdom Radiol (NY). 2024 May 11;49(9):3096–106. doi: 10.1007/s00261-024-04351-3 (PMC11335970; doi:10.1007/s00261-024-04351-3)
Supplement: Supplementary file 1 — Supplementary file1 (DOCX 307 KB) [file 261_2024_4351_MOESM1_ESM.docx]

**Interpretable multiphasic CT-based radiomics analysis for preoperatively differentiation benign and malignant solid renal tumors: a multi-centers study**

Supplementary Table 1 Contrast-enhanced CT protocols in the center1 and center 2

| Parameters | Center1 | | Center2 | |
| --- | --- | --- | --- | --- |
| Manufacturer | SIEMENS(N=265) | GE MEDICAL SYSTEMS(N=1) | Philips(N=46) | SIEMENS(N=115) |
| Scanner model | SOMATOM Definition Flash, SOMATOM Definition Edge | Revolution CT | IQon - Spectral CT | SOMATOM Definition Flash, SOMATOM Definition AS |
| Sequence | Axial | Axial | Axial | Axial |
| Thickness of slice (mm) | 3-7 | 5 | 3, 5 | 5 |
| Tube voltage (kV) | 100, 120,140 | 140 | 120 | 100, 120 |
| Tube current | 384-720mA (Automatic tube-current) | 200mA | 223-685mA (Automatic tube-current) | 112-600mA (Automatic tube-current) |
| Detector collimation (mm) | 64 × 0.6 | 64 × 0.625 | 64 × 0.625 | 64 × 0.6, 32 × 1.2, 16 × 1.2, |
| Gantry rotation time (s) | 0.5 | 0.5 | 0.4, 0.5 | 0.5 |
| Pitch | 1.2 | 0.984 | 1.11, 1.2, 1.258 | 0.6, 0.75, 0.8, 1, 1.2 |
| Matrix | 512 × 512 | 512 × 512 | 512 × 512 | 512 × 512 |
| Phase of contrast enhancement | Three phases | Three phases | Three phases | Three phases |
| Corticomedullary phase (s) | 25–35 | 25–35 | 25–35 | 25–35 |
| Nephrographic phase (s) | 70-90 | 70-90 | 70-90 | 70-90 |
| Excretory phase (s) | 160-180 | 160-180 | 160-180 | 160-180 |

Supplementary Table 2 Detailed histologic distribution of renal tumors in the training set and external validation set.

| Histologic type | | Training set | External validation set |
| --- | --- | --- | --- |
| Benign tumors | AML | 92 | 52 |
|  | RO | 3 | 1 |
| Malignant tumors | ccRCC | 148 | 89 |
|  | pRCC | 7 | 9 |
|  | chRCC | 16 | 10 |

Note: AML: angioleiomyolipoma, RO: renal oncocytoma, ccRCC: clear cell renal cell carcinoma, pRCC: papillary renal cell carcinoma, chRCC: chromophobe renal cell carcinoma

Supplementary Table 3 Detailed stages and grades of malignant renal tumors in the training set and external validation set.

| Characteristic | | Training set | External validation set |
| --- | --- | --- | --- |
| Participants | | 171 | 108 |
| Sex | Female | 65 | 36 |
|  | Male | 106 | 72 |
| Stage | I | 145 | 91 |
|  | II | 20 | 14 |
|  | III | 5 | 3 |
|  | IV | 1 | 0 |
| Grade | 1 | 20 | 10 |
|  | 2 | 90 | 63 |
|  | 3 | 29 | 17 |
|  | 4 | 7 | 1 |
|  | Not available | 25 | 17 |

Supplementary Table 4 Detailed distribution of T1a RCC and benign renal tumors of 4cm or less in the training set and external validation set.

| Set | Benign renal tumors | T1a RCC | Total |
| --- | --- | --- | --- |
| Training set | 38 | 90 | 128 |
| External validation set | 25 | 57 | 82 |

Supplementary Table 5 Selected features by the mRMR from CP, NP, and EP images

|  | selected 10 features |
| --- | --- |
| CP | original_shape_Flatness |
|  | wavelet-LLH_glrlm_LowGrayLevelRunEmphasis |
|  | wavelet-HHL_glcm_MCC |
|  | squareroot_firstorder_Skewness |
|  | squareroot_glcm_ClusterTendency |
|  | wavelet-HLH_glszm_GrayLevelNonUniformityNormalized |
|  | wavelet-HHH_glcm_SumSquares |
|  | exponential_glszm_LargeAreaHighGrayLevelEmphasis |
|  | square_glcm_Contrast |
|  | log-sigma-3-0-mm-3D_glszm_GrayLevelNonUniformityNormalized |
| NP | original_shape_Flatness |
|  | wavelet-HHL_glszm_ZoneVariance |
|  | square_glcm_ClusterProminence |
|  | wavelet-LHH_glrlm_ShortRunLowGrayLevelEmphasis |
|  | log-sigma-3-0-mm-3D_glszm_SmallAreaLowGrayLevelEmphasis |
|  | exponential_glrlm_RunLengthNonUniformity |
|  | original_ngtdm_Busyness |
|  | wavelet-HHH_firstorder_Variance |
|  | squareroot_firstorder_RobustMeanAbsoluteDeviation |
|  | original_shape_Sphericity |
| EP | original_shape_Flatness |
|  | wavelet-LLL_firstorder_Energy |
|  | exponential_glszm_GrayLevelNonUniformity |
|  | wavelet-HHL_glcm_ClusterProminence |
|  | gradient_firstorder_Minimum |
|  | wavelet-LHL_glcm_ClusterShade |
|  | wavelet-LHH_firstorder_Variance |
|  | square_firstorder_Minimum |
|  | square_glszm_LargeAreaEmphasis |
|  | logarithm_firstorder_RobustMeanAbsoluteDeviation |

Supplementary Table 6 The hyperparameters used to RF model construction in the train set

| Parameters | CP | NP | EP | TP |
| --- | --- | --- | --- | --- |
| bootstrap | TRUE | TRUE | TRUE | TRUE |
| ccp_alpha | 0 | 0 | 0 | 0 |
| criterion | gini | gini | gini | gini |
| max_depth | 3 | 7 | 7 | 11 |
| max_features | 3 | 3 | 1 | 3 |
| min_impurity_decrease | 0 | 0 | 0 | 0 |
| min_samples_leaf | 7 | 22 | 19 | 19 |
| min_samples_split | 104 | 101 | 50 | 44 |
| min_weight_fraction_leaf | 0 | 0 | 0 | 0 |
| n_estimators | 115 | 77 | 211 | 83 |
| oob_score | TRUE | TRUE | FALSE | TRUE |
| random_state | 66 | 1 | 66 | 66 |
| verbose | 0 | 0 | 0 | 0 |
| warm_start | FALSE | FALSE | FALSE | FALSE |

Supplementary Table 7 Performance of RF models for renal tumor patients with tumors ≤ 4cm in diameter in the training set and external validation set

| Phase | | AUC | ACC | Sen | Spe | PPV | NPV |
| --- | --- | --- | --- | --- | --- | --- | --- |
| Training set | CP | 0.844[0.781-0.906] | 0.805[0.736-0.873] | 0.889[0.834-0.943] | 0.684[0.604-0.765] | 0.870[0.811-0.928] | 0.722[0.645-0.800] |
|  | NP | 0.903[0.852-0.954] | 0.828[0.763-0.893] | 0.789[0.718-0.860] | 0.842[0.779-0.905] | 0.922[0.876-0.969] | 0.627[0.544-0.711] |
|  | EP | 0.898[0.845-0.950] | 0.766[0.692-0.839] | 0.856[0.795-0.916] | 0.763[0.690-0.837] | 0.895[0.842-0.948] | 0.690[0.610-0.771] |
|  | TP | 0.928[0.883-0.973] | 0.844[0.781-0.907] | 0.778[0.706-0.850] | 0.921[0.874-0.968] | 0.959[0.925-0.993] | 0.636[0.553-0.720] |
| External validation set | CP | 0.780[0.690-0.869] | 0.805[0.719-0.891] | 0.842[0.763-0.921] | 0.680[0.579-0.781] | 0.857[0.781-0.933] | 0.654[0.551-0.757] |
|  | NP | 0.742[0.647-0.836] | 0.756[0.663-0.849] | 0.877[0.806-0.948] | 0.560[0.453-0.667] | 0.820[0.736-0.903] | 0.667[0.565-0.769] |
|  | EP | 0.864[0.790-0.938] | 0.756[0.663-0.849] | 0.737[0.642-0.832] | 0.880[0.810-0.950] | 0.933[0.879-0.987] | 0.595[0.488-0.701] |
|  | TP | 0.829[0.748-0.911] | 0.768[0.677-0.860] | 0.895[0.828-0.961] | 0.680[0.579-0.781] | 0.864[0.790-0.939] | 0.739[0.644-0.834] |

Note. Data in square brackets are 95% CIs of corresponded values.


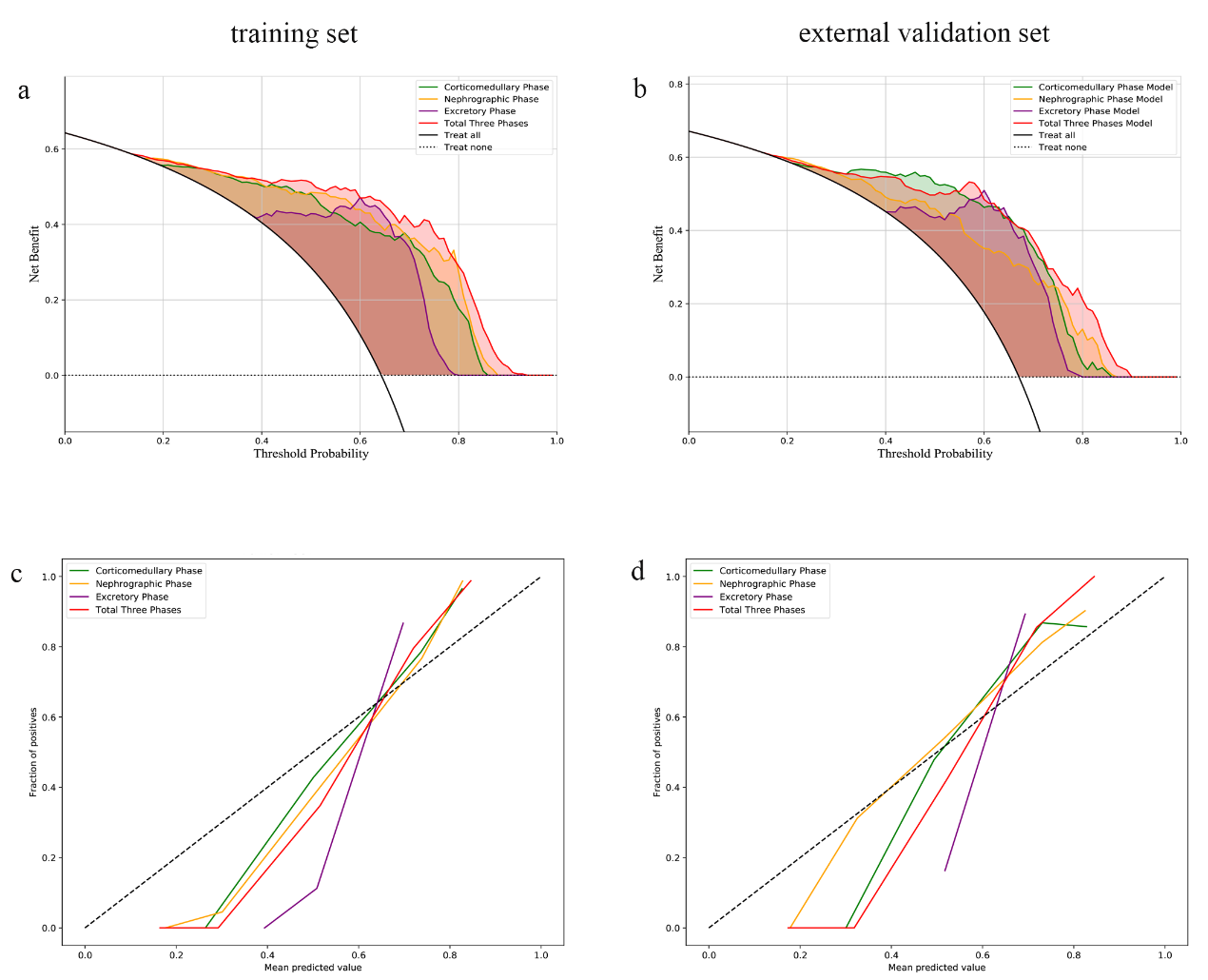


Supplementary Figure 1. DCA curve in the training set (a) and external test set (b), and calibration curve in the training set (c) and external test set (d) of RF models based on corticomedullary phase, nephrographic phase, excretory phase and total three phases for differentiation of benign from malignant renal tumors. RF= random forest.
